# IMPACT OF COVID ON PERSON AND FAMILY ENGAGEMENT IN ASSISTED LIVING

**DOI:** 10.1093/geroni/igac059.740

**Published:** 2022-12-20

**Authors:** Anna Beeber

**Affiliations:** Johns Hopkins University, Baltimore, Maryland, United States

## Abstract

Advancing person-centered care in assisted living (AL), while minimizing safety risks (e.g., injury, elopement, or medication errors), requires effective partnerships among residents, family members, and staff. The COVID-19 pandemic adversely affected capacity across and within AL settings to establish and nurture these relationships, ultimately affecting and changing person and family engagement in care. Using data from qualitative interviews, we will report findings about person and family engagement in the safety of AL from the perspectives of 104 residents, families, and staff. Particular attention will be given to examining the effect of COVID-19 on person and family engagement for residents at increased risk for disparities in assisted living, including residents who are living with mild cognitive impairment or dementia. The presentation highlights both challenges and promising practices that emerged from the COVID-19 pandemic. Implications for AL are presented to support the transition from pandemic to endemic.

